# Fatty Acid Synthase Is a Key Target in Multiple Essential Tumor Functions of Prostate Cancer: Uptake of Radiolabeled Acetate as a Predictor of the Targeted Therapy Outcome

**DOI:** 10.1371/journal.pone.0064570

**Published:** 2013-05-31

**Authors:** Yukie Yoshii, Takako Furukawa, Nobuyuki Oyama, Yoko Hasegawa, Yasushi Kiyono, Ryuichi Nishii, Atsuo Waki, Atsushi B. Tsuji, Chizuru Sogawa, Hidekatsu Wakizaka, Toshimitsu Fukumura, Hiroshi Yoshii, Yasuhisa Fujibayashi, Jason S. Lewis, Tsuneo Saga

**Affiliations:** 1 Molecular Imaging Center, National Institute of Radiological Sciences, Chiba, Japan; 2 Biomedical Imaging Research Center, University of Fukui, Fukui, Japan; 3 Faculty of Medical Sciences, University of Fukui, Fukui, Japan; 4 Department of Radiology, University of Miyazaki Hospital, Miyazaki, Japan; 5 Emergency Radiation Exposure Medical Care Research Center, National Institute of Radiological Sciences, Chiba, Japan; 6 Radiochemistry Service, Department of Radiology, Memorial Sloan-Kettering Cancer Center, New York, New York; Wayne State University, United States of America

## Abstract

Fatty acid synthase (FASN) expression is elevated in several cancers, and this over-expression is associated with poor prognosis. Inhibitors of FASN, such as orlistat, reportedly show antitumor effects against cancers that over-express FASN, making FASN a promising therapeutic target. However, large variations in FASN expression levels in individual tumors have been observed, and methods to predict FASN-targeted therapy outcome before treatment are required to avoid unnecessary treatment. In addition, how FASN inhibition affects tumor progression remains unclear. Here, we showed the method to predict FASN-targeted therapy outcome using radiolabeled acetate uptake and presented mechanisms of FASN inhibition with human prostate cancer cell lines, to provide the treatment strategy of FASN-targeted therapy. We revealed that tumor uptake of radiolabeled acetate reflected the FASN expression levels and sensitivity to FASN-targeted therapy with orlistat *in vitro* and *in vivo*. FASN-targeted therapy was noticeably effective against tumors with high FASN expression, which was indicated by high acetate uptake. To examine mechanisms, we established FASN knockdown prostate cancer cells by transduction of short-hairpin RNA against FASN and investigated the characteristics by analyses on morphology and cell behavior and microarray-based gene expression profiling. FASN inhibition not only suppressed cell proliferation but prevented pseudopodia formation and suppressed cell adhesion, migration, and invasion. FASN inhibition also suppressed genes involved in production of intracellular second messenger arachidonic acid and androgen hormones, both of which promote tumor progression. Collectively, our data demonstrated that uptake of radiolabeled acetate is a useful predictor of FASN-targeted therapy outcome. This suggests that [1-^11^C]acetate positron emission tomography (PET) could be a powerful tool to accomplish personalized FASN-targeted therapy by non-invasive visualization of tumor acetate uptake and selection of responsive tumors. FASN-targeted therapy could be an effective treatment to suppress multiple steps related to tumor progression in prostate cancers selected by [1-^11^C]acetate PET.

## Introduction

Fatty acid synthase (FASN) is a key enzyme in fatty acid synthesis from acetyl CoA, which is expressed at high levels in liver and adipose tissue, but at low levels in other tissues in humans [1]. FASN is over-expressed in several human cancers, including prostate, breast, lung, ovary, bladder, stomach, oral cavity and melanoma, and the over-expression is associated with poor prognosis [2], [3]. FASN has been demonstrated to play an important role in carcinogenesis by protecting cells from apoptosis [2].

In recent years, inhibitors of FASN reportedly show antitumor activity [4]. Orlistat, a selective inhibitor of FASN, is one of those poised for clinical use, particularly since orlistat is already used as an over-the-counter drug for obesity in the United States and European Union. Orlistat is reported to exert antitumor activity by inducing apoptosis in tumor cells [3], [5]. Thus, FASN-targeted therapy is expected to be effective against FASN-expressing tumors. However, large variations in FASN expression levels in individual tumors have been observed by pathological studies [2], [6]. Therefore, when considering application of FASN-targeted therapy, methods to predict therapeutic outcome in individual tumors before treatment are needed to reduce unnecessary treatment.

To approach this requirement, we focused on uptake of radiolabeled acetate in tumor cells. We have previously reported uptake mechanism of radiolabeled acetate into tumor cells; cytosolic acetyl-CoA synthetase, which converts acetate to acetyl-CoA, is over-expressed in tumor cells and plays a role in incorporation of radiolabeled acetate and the acetate incorporated into tumor cells is mainly used for fatty acid synthesis rather than breakdown to CO_2_ via tricarboxylic acid cycle or amino acid synthesis [7], [8]. Furthermore, Yun et al. 2009 have also shown that acetyl CoA synthetase is important in [1-^11^C]acetate uptake and linked with acetate-dependent fatty acid synthesis [9]. These evidences mean that acetate uptake mediated by acetyl CoA synthetase is associated with fatty acid synthesis in tumors. In fact, it has been reported that inhibitors of FASN, including orlistat, can reduce radiolabeled acetate incorporation into fatty acids in prostate cancer cells [5], [10]. Vāvere et al. have demonstrated that radiolabeled acetate uptake is correlated with FASN expression and [1-^11^C]acetate positron emission tomography (PET), which can non-invasively visualize uptake of acetate, is a useful tool to examine FASN expression levels in xenograft of prostate cancer cell lines [10]. These studies indicate that radiolabeled acetate uptake is a good surrogate marker of FASN expression in tumors. Therefore, radiolabeled acetate uptake may be a good predictor of FASN-targeted therapy outcome; however, it remains unclear whether outcome of FASN-targeted therapy can be predicted by uptake of radiolabeled acetate. In this study, we examined whether radiolabeled acetate uptake can predict therapeutic outcome of FASN-targeted therapy using human prostate cancer cell lines to demonstrate applicability of [1-^11^C]acetate PET as a predictor of FASN-targeted therapy outcome. So far, in clinical studies, [1-^11^C]acetate PET has been applied for the evaluation of various types of malignant tumor [11]–[14]; hence if the relationship between radiolabeled acetate uptake and FASN-targeted therapy outcome is proved, [1-^11^C]acetate PET could be promptly applied to predict FASN-targeted therapy outcome.

Additionally, the mechanisms how FASN inhibition affects tumor progression is not yet adequately addressed and therefore elucidation of the mechanisms is required to understand the significance of FASN inhibition and encourage utilization of FASN-targeted therapy. In this study, to investigate mechanisms, we established FASN knockdown prostate cancer cells by transduction of short-hairpin RNA (shRNA) against FASN, which can induce gene-specific silencing, and investigated the characteristics by analyses on morphology and cell behavior and microarray-based gene expression profiling. Through these studies, we discussed the new treatment strategy of FASN-targeted therapy in prostate cancer.

## Materials and Methods

### Cell Cultivation

Human prostate cancer cell lines, LNCaP (CRL-1740), PC3 (CRL-1435), 22Rv1 (CRL-2505), and DU145 (HTB-81) were obtained from American Type Culture Collection. Cells were incubated in a humidified atmosphere of 5% CO_2_ in air at 37°C. RPMI 1640 medium (Gibco, Carlsbad, CA, USA) supplemented with 10% fetal bovine serum and antibiotics was used for growth medium. Exponentially growing cells were used for experiments. Cells were trypsinized, and the number of viable cells was counted using the trypan blue dye-exclusion method.

### Cell Uptake Study of [1-^14^C]Acetate *In Vitro*

Cell uptake of [1-^14^C]acetate in LNCaP, PC3, 22Rv1, and DU145 cells was examined. Cells were seeded at 1 × 10^5^ in 1 ml of growth medium per well in 24-well plates and preincubated for 24 h before uptake study. After preincubation, medium was removed and 500 µl of growth medium containing 37 kBq of [1-^14^C]acetate (2.07 GBq/mmol) (GE Healthcare, Pollards, UK) was added to each well, and cells were incubated for 1 h at 37°C. Medium was then removed, and the cells were washed twice with ice-cold PBS. The resultant cells were lysed with 500 µl of 0.2 N NaOH for 2 h at room temperature, and the radioactivity of the lysate and liquid scintillator (ACSII; GE Healthcare) mixture was measured with a Tri-Carb liquid scintillation counter (PerkinElmer, Wallac, Turku, Finland). Cells treated in the same manner before cell lysis were counted using the trypan blue dye-exclusion method.

### Western Blot Analysis

FASN expression levels were examined by western blot analysis. Samples were lysed with lysis buffer containing protease inhibitor cocktail (Sigma, Saint Louis, MO, USA) and protein concentrations were determined by Bio-Rad protein assay kit (Bio-Rad, Hercules, CA, USA). SDS-polyacrylamide gel electrophoresis was performed with 20 µg of protein in each sample using 4%-15% mini-protean TGX precast gels (Bio-Rad). Rabbit anti-FASN primary antibody (sc-20140; Santa Cruz Biotechnology, Santa Cruz, CA, USA), rabbit anti-β-actin primary antibody (IMG-5142A; Imgenex, San Diego, CA, USA), and goat anti-rabbit IgG secondary antibody (G21234; Invitrogen, Carlsbad, CA, USA) were used. Bands were detected with the ECL Plus Western Blotting Detection System (GE Healthcare) and intensity was calculated by densitometry using Image J software (National Institutes of Health).

### Cell Viability after Orlistat Treatment

Effect of orlistat treatment *in vitro* was examined with LNCaP, PC3, 22Rv1, and DU145 cells. Cells were seeded at 1 × 10^4^ in 100 µl of growth medium per well in 96-well plates. After the preincubation for 24 h, medium was replaced with growth medium containing 0 µM, 12.5 µM, 25 µM, 50 µM, 250 µM or 500 µM orlistat (Cayman Chemical, Ann Arbor, MI, USA). The concentration of orlistat was decided based on the previous studies [3], [5]. Orlistat was dissolved in ethanol and added in the growth medium to contain less than 0.02% ethanol at final. After 48 h-incubation with medium containing orlistat, cell viability was analyzed by CellTiter-Glo luminescent cell viability assay (Promega, Fitchburg, WI, USA) according to the manufacturer’s protocol. CellTiter-Glo reagent was added directly to culture wells, and incubated for 10 min with shaking. Luminescence was recorded using a plate reader (SpectraMax M5, Molecular Devices, Sunnyvale, CA, USA). During the 48-h incubation with orlistat, the medium was refreshed at 24 h after the start of treatment.

### Implantation of Xenografts into Mice

This study was carried out in strict accordance with the recommendations in the Guide for the Care and Use of Laboratory Animals of the National Institute of Radiological Sciences (Japan). The protocol was approved by the Animal Ethics Committee of the National Institute of Radiological Sciences (Japan) (Permit Number: M40-01). NOD.CB17-Prkdc scid/J male mice (4 weeks of age) were obtained from Charles River Laboratories (Yokohama, Japan). Before the experiments, mice were kept undisturbed for at least 1 week. For *in vivo* studies, we used three representative cell lines in terms of FASN expression; LNCaP (high FASN expression), PC3 (low FASN expression), or DU145 (very low FASN expression). To obtain xenograft models, tumor cells at 3×10^7^ for LNCaP and 1×10^7^ for PC3 and DU145 were subcutaneously injected into mouse right shoulder with matrigel (BD Bioscience, Franklin Lakes, NJ, USA).

### *In Vivo* Biodistribution

For biodistribution study, mice with xenograft tumors were intravenously injected with 37 kBq [1-^14^C]acetate in 100 µl of saline into the tail vein and were euthanized at 10 min or 30 min after injection (4 mice per group). Organs of interest (tumor, muscle, lung, liver, spleen, pancreas, kidney and prostate) and blood were collected, and the weight of each organ was measured. Organs were digested in 1 ml of Soluene 350 (PerkinElmer) at 50°C for 2 h. Aliquots of hydrogen peroxide (30% w/v), 2 × 100 µL, were added sequentially to the samples to bleach. Scintillation medium (Hionic-Fluor, PerkinElmer) was added before counting the radioactivity in a Tri-Carb liquid scintillation counter (PerkinElmer). Biodistribution data were calculated as %ID/g (means ± SD).

### Small-Animal PET Imaging with [1-^11^C]Acetate

To confirm the biodistribution of uptake of radiolabeled acetate in xenograft models, small-animal PET imaging with [1-^11^C]acetate was performed. [1-^11^C]acetate was synthesized as previously reported [15]. Radiochemical purity was >99%. Dynamic PET scans of 60-min duration (12 × 5 min) was performed using a small animal PET system (Inveon, Siemens Medical Solutions, Malvern, PA, USA). Each mouse was intravenously injected with approximately 18.5 MBq of [1-^11^C]acetate via the tail vein under 1.5% isoflurane anesthesia. Body temperature was maintained by a lamp and heat pump during the scan. Images were reconstructed using a 3D maximum a posteriori using Inveon Acquisition Workplace software (Siemens Medical Solutions).

### *In Vivo* Orlistat Treatment

Therapeutic effects of orlistat were also investigated with tumor xenograft mice. Mice with well-established tumors (0.6–0.9-cm longest diameter) were randomized into orlistat-treatment and control groups (5 animals/group). Orlistat (240 mg/kg/day) was injected i.p. daily for 2 weeks. The treatment dose was determined based on previous studies [3], [5]. Orlistat was dissolved in 33 µl of ethanol, and diluted with 66 µl of saline just before injection. As a control, the equivalent amount of vehicle was injected in the same manner. Mice were weighed, and tumor sizes were measured using precision calipers 3 times weekly. Tumor volume was calculated using the equation: tumor volume = length×width^2^×π/6.

### RNA Interference (RNAi) Experiments

In this study, to investigate exact mechanisms how FASN inhibition affects tumor progression, we adopted FASN knockdown LNCaP cells established by transduction of shRNA against FASN, which can bring stable long-term gene-specific silencing [16]. FASN knockdown LNCaP cells were established by transduction of Mission lentiviral transduction particles (SHCLNV-NM 00410; Sigma) carrying expression cassettes encoding shRNAs that generate small-interfering RNAs, against FASN, according to the manufacturer’s protocol. Five clones of shRNAs against FASN (TRCN3125, 3126, 3127, 3128, and 3129) were used and cell lines transfected with each shRNA were established (designated FASN-RNAi 3125, 3126, 3127, 3128. and 3129 cells, respectively). Non-targeting shRNA (SHC002V, Sigma) was used as a negative control (control-RNAi cells). Cells (1 × 10^4^) were plated in 100 µl of growth medium per well on 96-well plates and incubated overnight. The medium was then changed to growth medium containing hexadimethrine bromide (Sigma) at a final concentration of 8 µg/ml to enhance transduction efficiency. Lentiviral particles (5–50 µl, 0.5–5 multiplicity of infection) were added and the plates were incubated overnight. Thereafter, we selected stable transductants expressing the shRNAs with puromycin. Expression levels of FASN mRNA were examined by qRT-PCR to check the efficiency of knockdown. For qRT-PCR, cell lysis, RNA isolation, reverse transcription, and real-time PCR were performed using the TaqMan Gene Expression Cells-to-CT kit (Applied Biosystems, Foster City, CA, USA) according to manufacturer’s protocol. Gene expression was analyzed using TaqMan Gene Expression Master Mix (Applied Biosystems) for β-actin (Hs99999903) and FASN (Hs00188012) with StepOne Real-Time PCR Systems (Applied Biosystems). FASN mRNA was quantified by the comparative C_T_ method using β-actin expression as an endogenous control [17].

### Cell Proliferation, Morphology, Migration, and Invasion

Cell behaviors were examined with FASN-RNAi 3128 and 3129 and control-RNAi LNCaP cells. For cell proliferation assay, cells were seeded onto 96-well plates at 5×10^3^ cells/100 µl of growth medium. After time course incubation, cell proliferation was determined by CellTiter-Glo luminescent assays. Cell morphology was observed using an inverted microscope (MDI 4000B; Leica, Solms, Germany). Time-lapse images were acquired every 2 h at 10× magnification for 5 days using a motorized inverted microscope (IX 81; Olympus, Tokyo, Japan) equipped with a stage top incubator (Tokai Hit, Fujinomiya, Japan) and data were analyzed with Fluoview software (Olympus). For migration and invasion assays, CytoSelect 96-well cell migration and invasion assays (Cell Biolabs, San Diego, CA, USA) were used according to the manufacturer’s protocol. Cells at 4×10^4^ cells/100 µl in serum-free media were placed in the top well and 150 µl of media containing 10% fetal bovine serum was placed in the bottom well, and cells were allowed to migrate or invade for 24 h before analysis. Top (non-migrating or non-invading) cells were removed, bottom (migrating or invading) cells were stained with dye solution and fluorescence was recorded using a plate reader at 480 nm/520 nm.

### DNA Microarray Analysis

Gene expression profiling was examined by DNA microarray analysis with FASN-RNAi 3128 and control-RNAi LNCaP cells. Total RNAs were isolated from cells with a Micro-to-Midi total RNA purification system (Invitrogen). The integrity of total RNAs was evaluated using an Agilent 2100 Bioanalyzer (Agilent Technologies, Santa Clara, CA, USA). Low Input Quick Amp Labeling Kit, one-color (Agilent Technologies) was used to prepare Cy3-labelled target cRNA according to the manufacturer’s instructions. Labeled cRNAs were hybridized with a SurePrint G3 Human GE 8×60K Microarrays (Agilent Technologies). Two separate hybridizations were performed for each sample. Array images were captured using a DNA Microarray Scanner (Agilent Technologies), and data were analyzed using Feature Extraction Software (Agilent Technologies) to obtain background-corrected signal intensities. Data were further analyzed with GeneSpring GX Software (Version 11.0, Agilent Technologies). After data filtering, mRNAs differentially expressed in target cells versus controls were assessed by Fisher’s exact test, followed by multiple corrections using the Benjamini and Hochberg false discovery rate (FDR) method. Gene sets with a FDR *q*-value <0.05 were considered to be significant. DNA microarray data can be found in the Gene Expression Omnibus database under accession number (GSE39183).

### Statistical Analysis

Data are expressed as means ± SD. *P* values were calculated by analysis of variance (ANOVA) for comparison between multiple treatment groups. Statistical analysis of tumor volumes was performed by repeated ANOVA, followed by post-hoc Tukey’s test. *P* values <0.05 were considered to be statistically significant.

## Results

### *In Vitro* Studies

**Figure 1:**
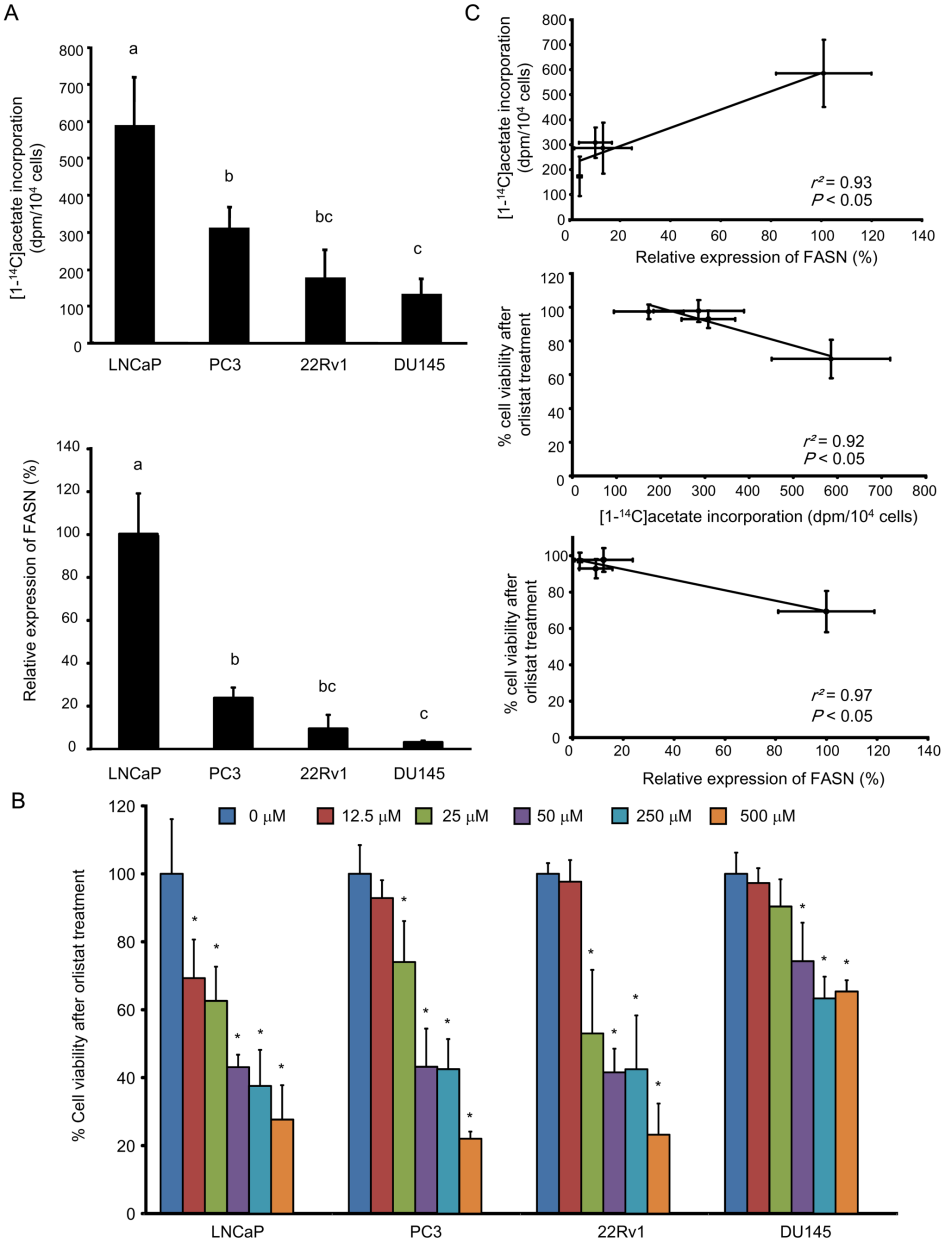
Relationships between uptake of radiolabeled acetate, FASN expression, and sensitivity to orlistat treatment *in vitro*. (A) Uptake of [1-^14^C]acetate (upper) and levels of FASN expression (lower) in LNCaP, PC3, 22Rv1, and DU145 cells. The groups with different alphabets are significantly different (*P*<0.05). (B) Percent cell viability after orlistat treatment in LNCaP, PC3, 22Rv1, and DU145 cells. Cell viability is indicated as a percentage of that with 0 µM orlistat treatment. Asterisks indicate statistical significance versus treatment with 0 µM for each cell line (*P<0.05). (C) Relationship between uptake of [1-^14^C]acetate and FASN expression (upper), relationship between % cell viability after orlistat treatment at 12.5 µM and uptake of [1-^14^C]acetate (middle), and relationship between % cell viability after orlistat treatment at 12.5 µM and FASN expression (lower). Values from six independent experiments are shown. Data are expressed as means ± SD.

We examined relationships between uptake of radiolabeled acetate, FASN expression, and sensitivity to orlistat treatment *in vitro* with LNCaP, PC3, 22Rv1, and DU145 cell lines (Fig. 1). [1-^14^C]acetate uptake after 1 h is shown in Fig. 1A, upper. High uptake of [1-^14^C]acetate was observed in LNCaP cells, while that in PC3 and 22Rv1 was lower, and that in DU145 was very low (*P*<0.05). FASN expression showed a trend complementing that observed in the uptake of [1-^14^C]acetate (Fig. 1A, lower): LNCaP cells showed higher FASN expression compared to the other cell lines (*P*<0.01). We confirmed that there was a strong positive correlation between uptake of [1-^14^C]acetate and FASN expression (*R^2^* = 0.93, *P*<0.05) (Fig. 1C, upper).

Fig. 1B shows % cell viability after orlistat treatment *in vitro*. Under low dose of orlistat treatment at 12.5 µM, LNCaP cells, which had shown the highest uptake of [1-^14^C]acetate and FASN expression, showed a significant decrease in % cell viability (*P*<0.05), but there were no significant decreases in % cell viability in PC3, 22Rv1, and DU145 cells. With 25 µM orlistat treatment, besides LNCaP cells, PC3 and 22Rv1 cells, which had shown relatively low uptake of [1-^14^C]acetate and FASN expression, showed a significant decrease in % cell viability, while DU145 cells, which had shown the lowest uptake of [1-^14^C]acetate and FASN expression, showed no decrease in viability. With over 50 µM orlistat treatment, all cell lines examined showed a significant decrease in % cell viability, but the changes in DU145 cells were moderate. There was a significant negative correlation between % cell viability, and uptake of [1-^14^C]acetate and FASN expression, respectively (*R^2^* = 0.92, *P*<0.05; *R^2^* = 0.97, *P*<0.05), under low-dose orlistat treatment at 12.5 µM (Fig. 1C, middle and lower). Whereas, there was no significant correlation between % cell viability, and uptake of [1-^14^C]acetate and FASN expression, respectively, under high-dose orlistat treatment. These data demonstrated higher sensitivity to orlistat treatment in the cells with higher FASN expression and uptake of acetate.

### Biodistribution and [1-^11^C]Acetate PET Study with Tumor-Bearing Mice

**Figure 2:**
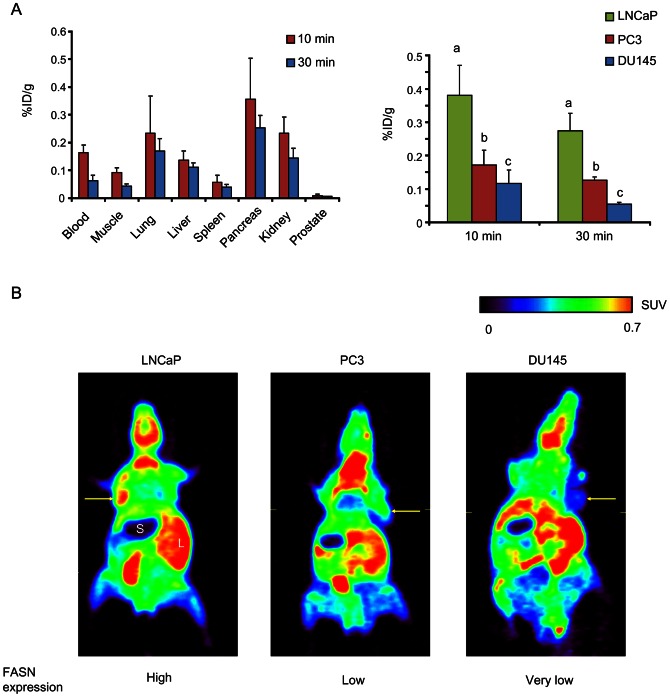
Biodistribution of [1-^14^C]acetate and *in vivo* PET imaging with [1-^11^C]acetate in tumor xenograft-bearing mice. (A) Biodistribution at 10 min and 30 min in organs (left) and each tumor (right). Data represents %ID/g, expressed as means ± SD. The groups with different alphabets are significantly different (*P*<0.05). (B) Small-animal PET images of [1-^11^C]acetate at 30 min after injection. Yellow arrows indicate tumors. S = stomach; L = liver.

For *in vivo* studies, we used mice bearing LNCaP (high FASN expression), PC3 (low FASN expression), or DU145 (very low FASN expression) tumors. Levels of FASN expression of each tumor xenograft was confirmed prior to the experiment, which showed similar tendency to the *in vitro* study (Fig. S1). First, to investigate absolute uptake of radiolabeled acetate in these xenograft tumors, we performed biodistribution study. Biodistribution data were obtained at 10 min and 30 min after injection of [1-^14^C]acetate (Fig. 2A). Time points for sampling were decided based on a previous study [10]. Biodistribution in the blood and normal organs showed a similar pattern as in previous reports [18]. [1-^14^C]acetate was cleared from the blood at 30 min, as compared with 10 min (Fig. 2A, left). At 30 min, LNCaP tumors (0.27±0.05%ID/g) showed 2.2-fold higher uptake of [1-^14^C]acetate than PC3 tumors (0.13±0.01%ID/g; *P*<0.01) and 5.5-fold higher uptake than DU145 tumors (0.06±0.01%ID/g; *P*<0.001) (Fig. 2A, right). Tumor-to-blood and tumor-to-muscle ratios at 30 min were also higher in LNCaP tumors compared with the PC3 and DU145 tumors (Fig. S2). Next, small animal PET with [1-^11^C]acetate was performed to confirm and supplement the results of biodistribution study. As the images demonstrated, [1-^11^C]acetate showed clear tumor accumulation in LNCaP xenograft model, while moderate or low accumulation of [1-^11^C]acetate was observed in PC3 and DU145 xenograft model (Fig. 2B). Therefore, biodistribution and [1-^11^C]acetate PET studies demonstrated that uptake of radiolabeled acetate reflects FASN expression levels and can distinguish tumors with high FASN expression from tumors with low FASN expression.

### FASN-Targeted Therapy *In Vivo*

**Figure 3:**
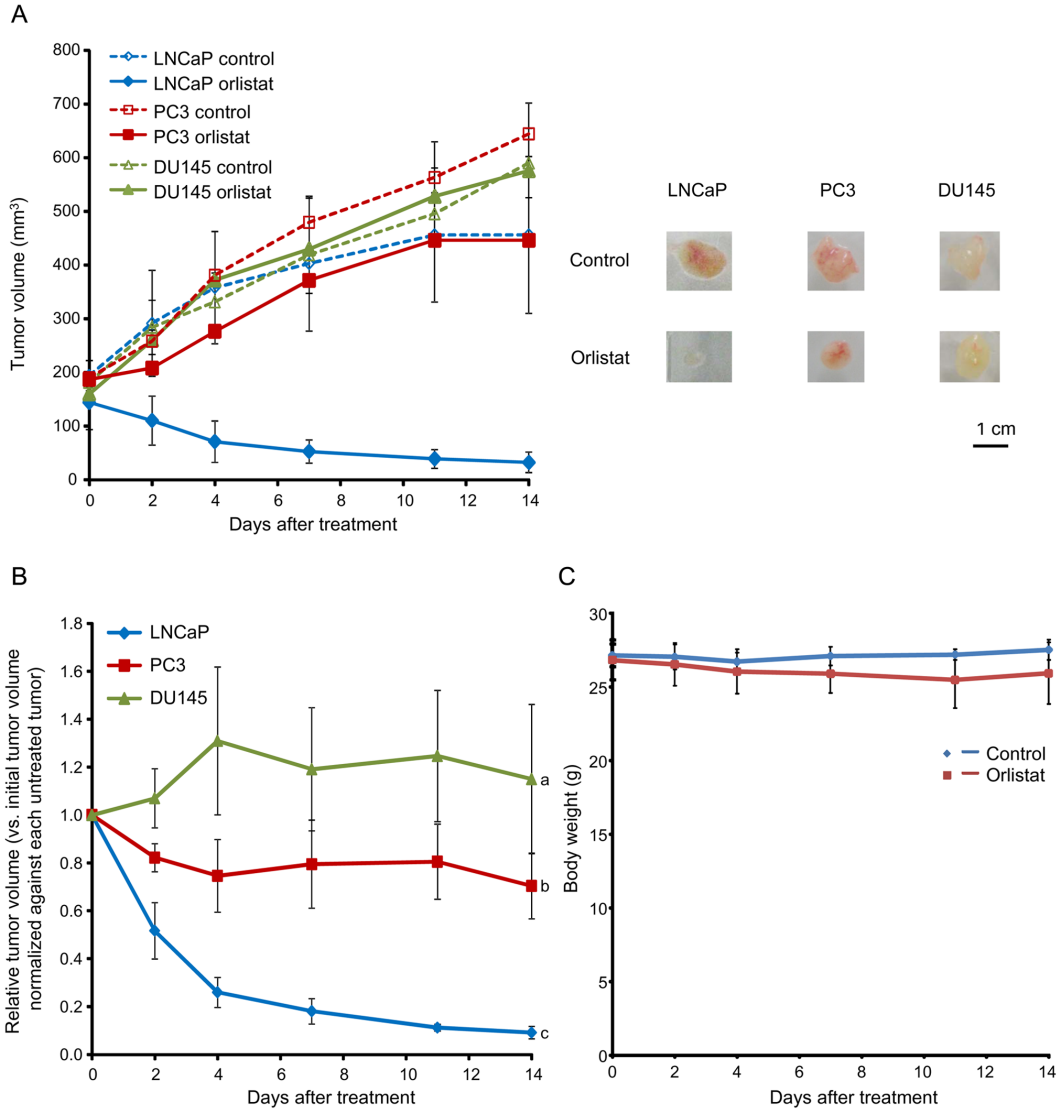
Effects of orlistat treatment *in vivo*. (A) Growth of tumors treated with orlistat (240 mg/kg/day) or vehicle only daily for 2 weeks (left) in tumor xenograft-bearing mice. Data represents tumor volume (mm^3^). Representative images of treated tumors at day 14 (right). (B) Relative tumor volume versus initial tumor size normalized against each untreated tumor at the same time point. The groups with different alphabets are significantly different (*P*<0.05). (C) Effects of orlistat treatment on body weight.

We further examined sensitivity of FASN-targeted therapy with orlistat in each tumor xenograft *in vivo* (Fig. 3). Fig. 3A shows tumor volume measurement. At day 14, tumor volume in LNCaP tumors treated with orlistat had decreased markedly; 22.0±6.2% of initial tumor volume. In contrast, PC3 and DU145 tumors treated with orlistat showed progressive increases in tumor volume; 238.8±46.6% and 367.1±99.5% of initial tumor volume, respectively. Fig. 3B shows tumor volume in LNCaP, PC3, and DU145 tumors treated with orlistat, shown in terms of relative tumor volume versus initial tumor size normalized against each untreated tumor at the same time point; there were significant differences in tumor growth between LNCaP, PC3, and DU145 tumors (*P*<0.05). The effects of orlistat treatment on body weight are shown in Fig. 3C. Body weight loss was less than 5% at day 14. There were no apparent changes in physical condition (with no sign of scruffy coat or diarrhea) over the experimental period. These data demonstrated that uptake of radiolabeled acetate reflects sensitivity of FASN-targeted therapy with orlistat *in vivo* and FASN-targeted therapy with orlistat is highly effective against tumors with high FASN expression indicated by high uptake of radiolabeled acetate.

### FASN Inhibition by RNAi

**Figure 4:**
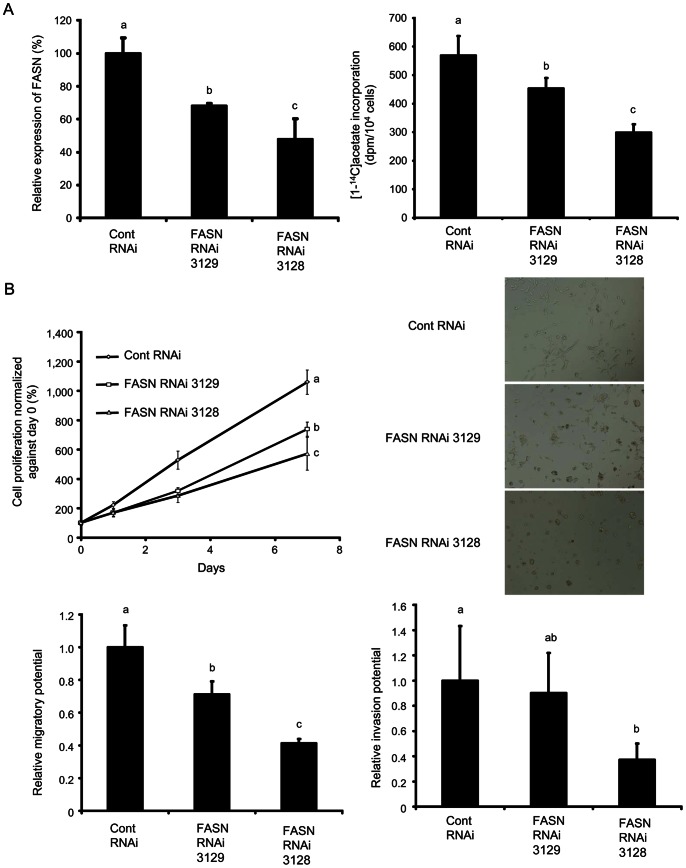
Effects of FASN inhibition by RNAi in FASN-expressing LNCaP cells. FASN-RNAi 3128 and 3129 cells and control-RNAi cells were used. (A) Relative expression of FASN, analyzed by Western blotting analysis (left) and uptake of [1-^14^C]acetate (right). (B) Cell proliferation over 7 days (upper, left). Light microscopy images (upper, right). Relative migration and invasion potential in FASN-RNAi cells, as compared with control-RNAi cells (lower, left and right, respectively). Values from six independent experiments are shown. Data are expressed as means ± SD. The groups with different alphabets are significantly different (*P*<0.05).

Next, to examine the mechanisms how FASN inhibition affects tumor progression, we adopted FASN knockdown LNCaP cells obtained by shRNA transduction. In this study, we established five FASN-RNAi cell lines, FASN-RNAi 3125, 3126, 3127, 3128, and 3129 cells, and qRT-PCR showed a marked decrease in FASN mRNA expression in FASN-RNAi 3128 cells and a moderate decrease in FASN-RNAi 3129 cells, as compared with control-RNAi cells (Fig. S3). Western blot analysis showed the same trend in FASN-RNAi 3128 and 3129 cells in FASN expression, respectively (Fig. 4A, left). Knockdown of FASN by RNAi in LNCaP cells led to a corresponding reduction in acetate incorporation (Fig. 4A, right).

**Figure 5:**
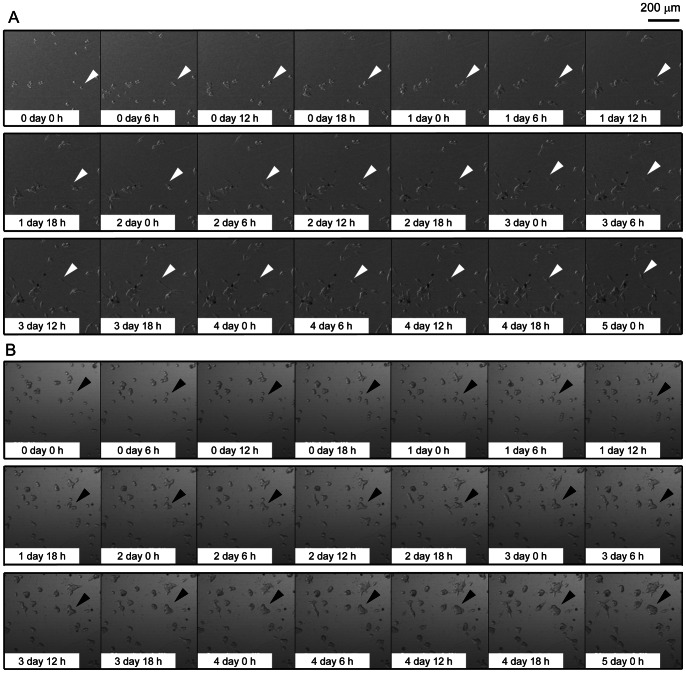
Time-lapse analysis showing morphological changes and movement of FASN knockdown LNCaP cells. Data indicate images in control-RNAi cells (A) and FASN-RNAi 3128 cells (B). Images were taken every 6 h for 5 days. White arrowheads indicate an example of the formation of pseudopodia, spindle-shaped morphology, and active cell migration in control-RNAi cells. Black arrowheads indicate an example of the deficient formation of pseudopodia, round morphology, and low activity of cell migration in the FASN-RNAi 3128 cells.

Interestingly, the knockdown of FASN induces observable changes in the biological characteristics of LNCaP cells (Fig. 4B). Knockdown of FASN inhibited cellular proliferation corresponding to a decrease in FASN expression (Fig. 4B, upper left); there were significant differences in cell proliferation in FASN-RNAi 3128 and 3129 cells, as compared to control-RNAi cells (*P*<0.05). Furthermore, microscopic observation showed that control-RNAi cells were spindle-shaped with pseudopodia on culture plates, while FASN-RNAi 3128 cells were round with deficient formation of pseudopodia and FASN-RNAi 3129 cells showed intermediate morphology (Fig. 4B, upper right). The movement of the cells on culture plates was further observed using time-lapse microscopy (Fig. 5, Video S1, Video S2). The control-RNAi cells attached to the plate bottom formed pseudopodia, became spindle-shaped, and actively migrated with cell division and elongation of their pseudopodia. While, FASN-RNAi 3128 cells showed deficient pseudopodia formation, became round, and the cells divided but showed less migration compared with control-RNAi cells. FASN-RNAi 3129 cells showed intermediate behaviors (data not shown).

Based on these observations, we hypothesize that FASN inhibition affects the migration and invasion of cells. To test this, we performed cell migration and invasion assays with the FASN-RNAi and control-RNAi LNCaP cells. We found that the FASN-RNAi cells showed less migration and invasion corresponding to decreases in the expression of FASN (Fig. 4B, lower left and right). These observations indicate that FASN inhibition not only suppressed cellular proliferation, but also impaired cellular adhesion, migration, and invasion.

### Gene Expression Profile in FASN Knockdown Cells

To further understand effects of FASN inhibition, gene expression profiling was performed with FASN-RNAi 3128 and control-RNAi LNCaP cells. Genes significantly down-regulated to less than half and up-regulated to more than 2-fold by FASN inhibition with RNAi (*P*<0.05), as compared with controls, are listed in Table 1 and Table S1, respectively. In Table 1, the expression of genes related to cellular proliferation [phospholipase A2, group IVA (PLA2G4A), tensin 3 (TNS3), and glypican 4 (GPC4)], cell adhesion and extracellular matrix organization [peroxidasin homolog (Drosophila) (PXDN), sarcoglycan epsilon (SGCE), von Willebrand factor (VWF), hydroxysteroid (17-beta) dehydrogenase 12 (HSD17B12), and cysteine-rich secretory protein LCCL domain containing 2 (CRISPLD2)], and cell motility [TNS3 and a member of the RAS oncogene family (RAP2B)] were shown to be down-regulated by FASN inhibition. FASN inhibition led to down-regulation of PLA2G4A and HSD17B12 genes, encoding phospholipase A2 and17-β hydroxysteroid dehydrogenase, respectively, which are key enzymes in production of intracellular second messenger arachidonic acid and androgen hormones, both playing roles in promotion of tumor progression [19], [20]. We also found that the genes related to arachidonic acid signaling, including regulator of G-protein signaling 2 (RGS2), sperm associated antigen 16 (SPAG16), VWF, and RAP2B, were suppressed with FASN inhibition [21]–[24]. Additionally, FASN inhibition resulted in down-regulation of many genes reported as valuable diagnostic and therapeutic targets in tumors (indicated in Table 1) [25]–[34]. Gene expression profiling therefore demonstrated that FASN inhibition induces down-regulation of genes related to cell proliferation, cell adhesion, migration, and invasion, as well as production of arachidonic acid and androgen hormones. Furthermore, in Table S2, up-regulation of genes related to induction of apoptosis, including thrombospondin 1 (THBS1), ras homolog gene family, member B (RHOB), pleckstrin homology-like domain, family A, member 2 (PHLDA2), and reticulon 4 (RTN4) [35]–[38], was observed, which supported the previous findings that FASN inhibition induces apoptosis [3], [5].

**Table 1:** Genes down-regulated by FASN inhibition with RNAi*.

Gene Symbol	Gene Name	Fold change	*P*-value	Biological process
PXDN†	peroxidasin homolog (Drosophila)	0.002	3E-05	Extracellular matrix organization
SGCE	sarcoglycan, epsilon	0.023	1E-06	Cell adhesion
CD302	CD302 molecule	0.028	2E-06	
MT1G	metallothionein 1G	0.06	2E-05	
DPYSL3	dihydropyrimidinase-like 3	0.066	2E-05	
SPAG16†	sperm associated antigen 16	0.091	2E-05	
RGS2†	regulator of G-protein signaling 2, 24kDa	0.099	1E-04	
PLA2G4A†	phospholipase A2, group IVA (cytosolic, calcium-dependent)	0.135	1E-05	Cell proliferation
TNS3†	tensin 3	0.149	1E-04	Cell proliferation, Cell motility
KCNE2	potassium voltage-gated channel, Isk-related family, member 2	0.202	3E-05	
RAPGEF5	Rap guanine nucleotide exchange factor (GEF) 5	0.209	9E-05	
CYP2S1	cytochrome P450, family 2, subfamily S, polypeptide 1	0.238	1E-04	
HTATIP2	HIV-1 Tat interactive protein 2, 30kDa	0.246	6E-05	
PAX1	paired box 1	0.252	1E-04	
SOX11	SRY (sex determining region Y)-box 11	0.257	2E-05	
RAP2B†	RAP2B, member of RAS oncogene family	0.287	1E-04	Cell motility
HS6ST2	heparan sulfate 6-O-sulfotransferase 2	0.302	2E-04	
SORBS2	sorbin and SH3 domain containing 2	0.334	1E-04	
VWF†	von Willebrand factor	0.346	3E-05	Cell adhesion
GPC4†	glypican 4	0.363	1E-04	Cell proliferation
ADH1C†	alcohol dehydrogenase 1C (class I), gamma polypeptide	0.405	3E-06	
HSD17B12†	hydroxysteroid (17-beta) dehydrogenase 12	0.44	5E-05	Extracellular matrix organization, Cell adhesion
CRISPLD2	cysteine-rich secretory protein LCCL domain containing 2	0.492	2E-04	Extracellular matrix organization

* Genes down-regulated by FASN inhibition in cells treated with FASN-RNAi 3128 in DNA microarray analysis. Genes significantly down-regulated to less than 0.5-fold (*P*<0.05) are listed.

† Genes reported as a potential tumor diagnostic or therapeutic target [25]–[34].

## Discussion

In this study, we demonstrated that uptake of radiolabeled acetate reflects FASN expression and sensitivity to FASN-targeted therapy, indicating uptake of radiolabeled acetate is a useful predictor of FASN-targeted therapy outcome. Furthermore, we discovered that FASN inhibition suppressed cell proliferation as well as cell adhesion, migration, and invasion and FASN inhibition led to suppression of genes involved in production of arachidonic acid and androgen hormones, both of which promote tumor progression. These findings indicate that FASN is a key target to suppress multiple steps that are important in tumor progression.

The overexpression of FASN is observed in several types of human cancer and this overexpression is reportedly associated with poor prognosis [2], [3]. In this study, we focused on prostate cancer, which is the second leading cause of cancer death for males in the United States [39]. Overexpression of FASN is also present in prostate cancers and the FASN expression is related to carcinogenesis, growth, and metastasis, while large variation in FASN expression among individual prostate cancers has been reported [2], [6]. Rossi et al. examined FASN expression levels in primary prostate cancers from 64 patients and found that FASN expression was strong in 8%, moderate in 30%, and weak in 53%, with 9% showing no expression [6]. Migita et al. showed that, from analysis with prostate cancer specimens from 745 patients, the FASN expression varies among individual tumors and is associated with anti-apoptotic activity [2]. Hence, FASN is expected to be a good therapeutic target for prostate cancer. Because of the frequency and variation of FASN overexpression in prostate cancer, it is necessary to predict therapeutic outcomes before application of FASN-targeted therapy to avoid ineffective treatment. In this study, we demonstrated that uptake of radiolabeled acetate can predict therapeutic outcome of the FASN-targeted therapy in prostate cancer; it was particularly successful for prostate cancer with high acetate uptake. Taken together with the fact that FASN inhibition can suppress multiple important steps in tumor progression, FASN-targeted therapy can be an effective treatment if uptake of radiolabeled acetate indicates subject tumors to be “responsive”.

**Figure 6:**
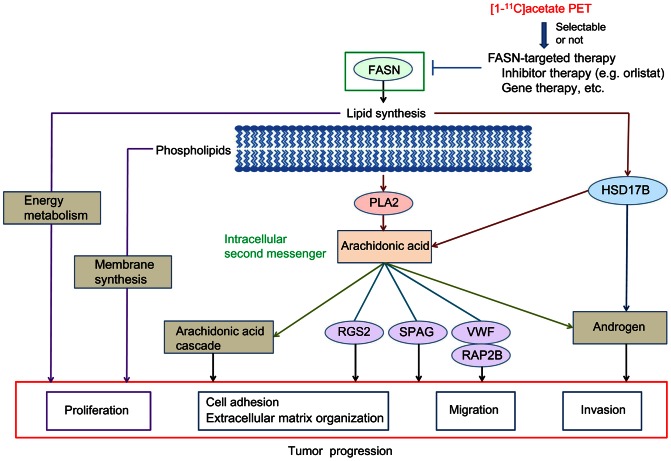
Schematic view of this study. The functions of FASN in tumors, mechanism of FASN inhibition, and method for applying [1-^11^C]acetate PET as a predictor of outcome of FASN-targeted therapies are shown.

It has been so far reported that FASN produces fatty acids as essential constituents of membrane phospholipids and important substrates for energy metabolism and that FASN inhibition can induce decrease of the lipid production in tumor cells [5], [40], [41]. Based on these evidences, it is assumed that FASN inhibition would cause lipid starvation and membrane dysfunction [40]. In this study, we discovered that FASN inhibition prevented pseudopodia formation and suppressed cell adhesion, migration, and invasion in prostate cancer cells. Pseudopodia reportedly play critical roles in the control of cell structure, cell adhesion, migration, and invasion, which are key steps related to tumor proliferation and metastasis [42]. From these evidences and our data, it is speculated that the lipid starvation and membrane dysfunction caused by FASN inhibition could lead to reduced cell proliferation, as well as deficient formation of pseudopodia, triggering suppression of cell adhesion, migration, and invasion that drive tumor progression (Fig. 6).

Furthermore, FASN inhibition led to down-regulation of PLA2G4A and HSD17B12 genes encoding phospholipase A2 and17-β hydroxysteroid dehydrogenase, respectively, which are key enzymes related to production of arachidonic acid and androgen hormones [19], [20] (Table 1). Phospholipase A2 is involved in release of arachidonic acid from phospholipids produced via fatty acid synthesis by FASN [19]. 17-β hydroxysteroid dehydrogenase is involved in arachidonic acid production by elongation of very long chain fatty acid [20]. Arachidonic acid is known to act as an intracellular second messenger that induces androgen production and promotes tumor progression [43]. Arachidonic acid is also involved in cellular proliferation, neo-angiogenesis, and metastasis through activation of cyclooxygenase-2, 5-lipoxygenase, and cytochrome P450 epoxygenase in arachidonic acid cascade [44]. Additionally, our data indicated that the genes related to the arachidonic acid signaling, including RGS2, SPAG16, VWF, and RAP2B [21]–[24], were also suppressed by FASN inhibition. Furthermore, 17-β hydroxysteroid dehydrogenase is known to participate in biosynthesis of androgen hormone [20] and the androgen production in cancer cells by 17-β hydroxysteroid dehydrogenase is also reported to play a critical role in development of human prostate cancer [45]. Taken together, these data suggest an additional mechanism of FASN inhibition; that is, FASN inhibition disrupts tumor progression via suppression of arachidonic acid and androgen production (Fig. 6). This may be because FASN inhibition is able to suppress synthesis of fatty acids as raw materials to produce arachidonic acid.

In this study, we preclinically demonstrated that uptake of radiolabeled acetate can predict FASN-targeted therapy outcome. [1-^11^C]acetate PET, which can non-invasively visualize tumor uptake of acetate, has been already applied for clinical studies [11]–[14]. Therefore, it is considered that [1-^11^C]acetate PET would be promptly applicable as a predictor of FASN-targeted therapy outcome in clinical settings. Then, [1-^11^C]acetate PET could be an effective tool to accomplish personalized FASN-targeted therapy for patients with prostate cancer by providing information to select those who would be benefitted by the FASN-targeted therapy (Fig. 6).

Clinically, anti-androgenic hormone therapy is a mainstay of treatment against prostate cancer, because growth of prostate cancer is dependent on androgen at early stages of treatment. Anti-androgen hormone therapy alone does not fully cure prostate cancer and prolonged therapy often results in relapse through conversion to androgen-independent cancer [46]. Hence, curative treatment at early stages of prostate cancer is important. Previous pathological studies have shown that FASN overexpression is strongly associated with prostate cancer progression and metastasis [6]. Intriguingly, this study indicates that FASN inhibition is able to suppress multiple steps that are important in tumor progression and metastasis, such as cell proliferation, cell adhesion, migration, and invasion, and that FASN inhibition is also able to suppress arachidonic acid and androgen production. This means that FASN inhibition can provide multipronged benefits for treatment, by suppressing tumor progression and metastasis, as well as preventing androgen-dependent growth in prostate cancers. Therefore, FASN-targeted therapy would be an effective treatment for complementing hormone therapy against FASN-positive prostate cancers with aggressive features and [1-^11^C]acetate PET could be applied to select the patients who can undergo the FASN-targeted therapy.

So far, it is known that [1-^11^C]acetate PET is useful for the clinical diagnostic investigation in brain tumors and hepatocellular carcinoma besides prostate cancer [47]. In brain tumors and hepatocellular carcinoma, it has been also reported that FASN is highly expressed and FASN inhibition is effective to reduce cell proliferation and that radiolabeled acetate uptake is associated with the fatty acid synthesis [48]–[50]. Therefore, FASN-targeted therapy would be effective and [1-^11^C]acetate PET might be useful as a predictor of FASN-targeted therapy outcome in brain tumors and hepatocellular carcinoma as well as in prostate cancer. Further study would be necessary to expand the applicability of this method.

### Conclusions

This study demonstrated that uptake of radiolabeled acetate is able to predict outcome of FASN-targeted therapy and that FASN inhibition not only suppressed cell proliferation but also prevented pseudopodia formation and suppressed cell adhesion, migration, and invasion, with human prostate cancer cell lines. Our data suggest that [1-^11^C]acetate PET could be a powerful tool for judging selectability of FASN-targeted therapy in individual patients in the clinic. Also, FASN-targeted therapy could be an effective treatment to suppress multiple tumor essential functions involved in tumor progression and metastasis in prostate cancers selected by [1-^11^C]acetate PET. Thus, this study provides the new treatment strategy of FASN-targeted therapy in prostate cancer.

## Supporting Information

Figure S1 **Levels of FASN expression of tumor xenograft in mice (LNCaP, PC3, and DU145).**
(TIF)

Figure S2 **Tumor-to-blood and tumor-to-muscle ratios in biodistribution study.**
(TIF)

Figure S3 **FASN mRNA expression in LNCaP cell lines transfected with shRNA against FASN.**
(TIF)

Table S1 **Genes up-regulated by FASN inhibition with RNAi.**
(DOC)

Video S1 **Video of morphological changes and movement of control RNAi cells obtained by time-lapse analysis.**
(WMV)

Video S2 **Video of morphological changes and movement of FASN RNAi 3128 cells obtained by time-lapse analysis.**
(WMV)
