# Supplementary figures and images for: Fatty Acid Synthase Is a Key Target in Multiple Essential Tumor Functions of Prostate Cancer: Uptake of Radiolabeled Acetate as a Predictor of the Targeted Therapy Outcome
Source: PLoS One. 2013 May 31;8(5):e64570. doi: 10.1371/journal.pone.0064570 (PMC3669310; doi:10.1371/journal.pone.0064570)

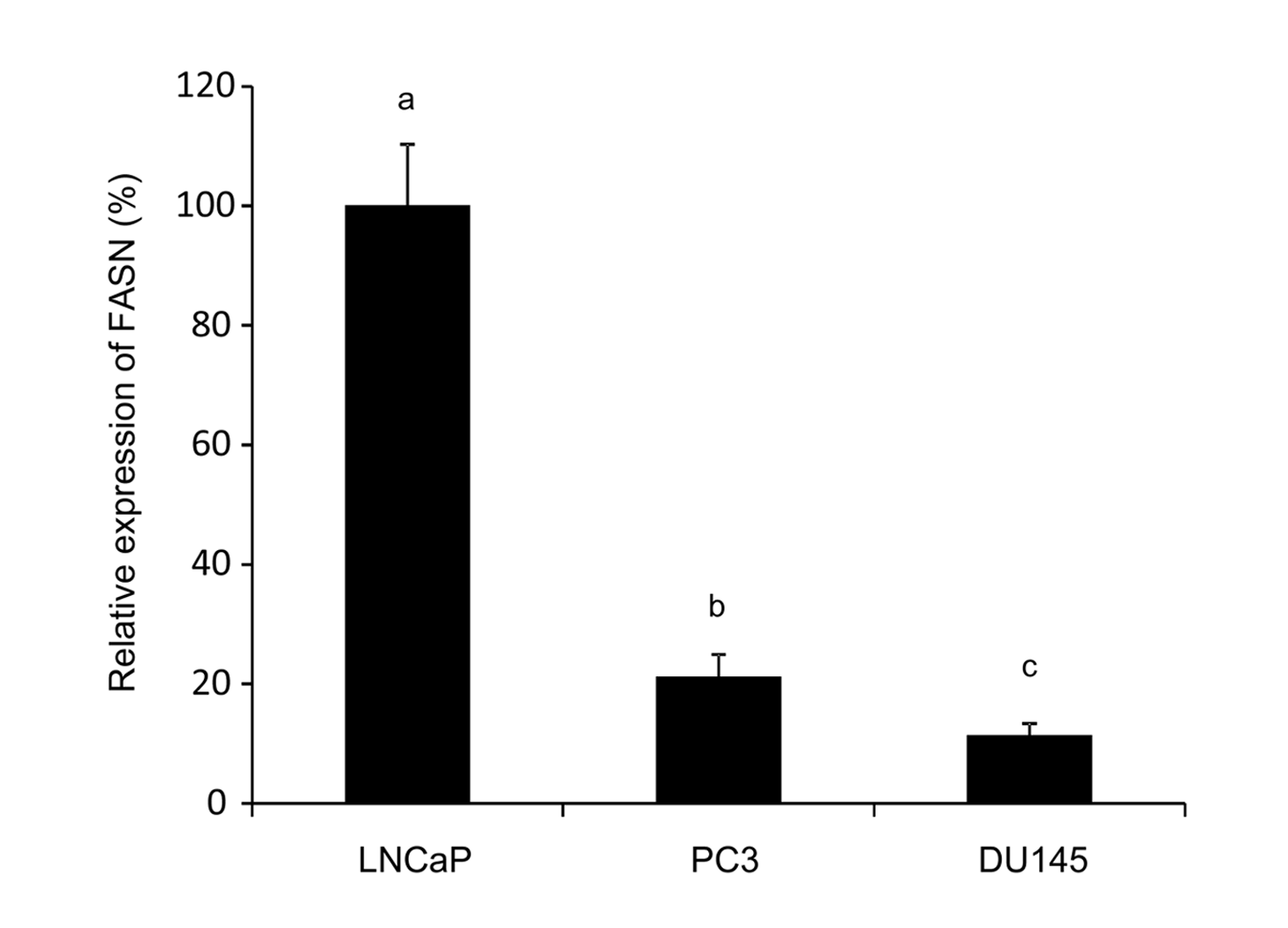

Supplement: Figure S1 — Levels of FASN expression of tumor xenograft in mice (LNCaP, PC3, and DU145). (TIF) [file pone.0064570.s001.tif]

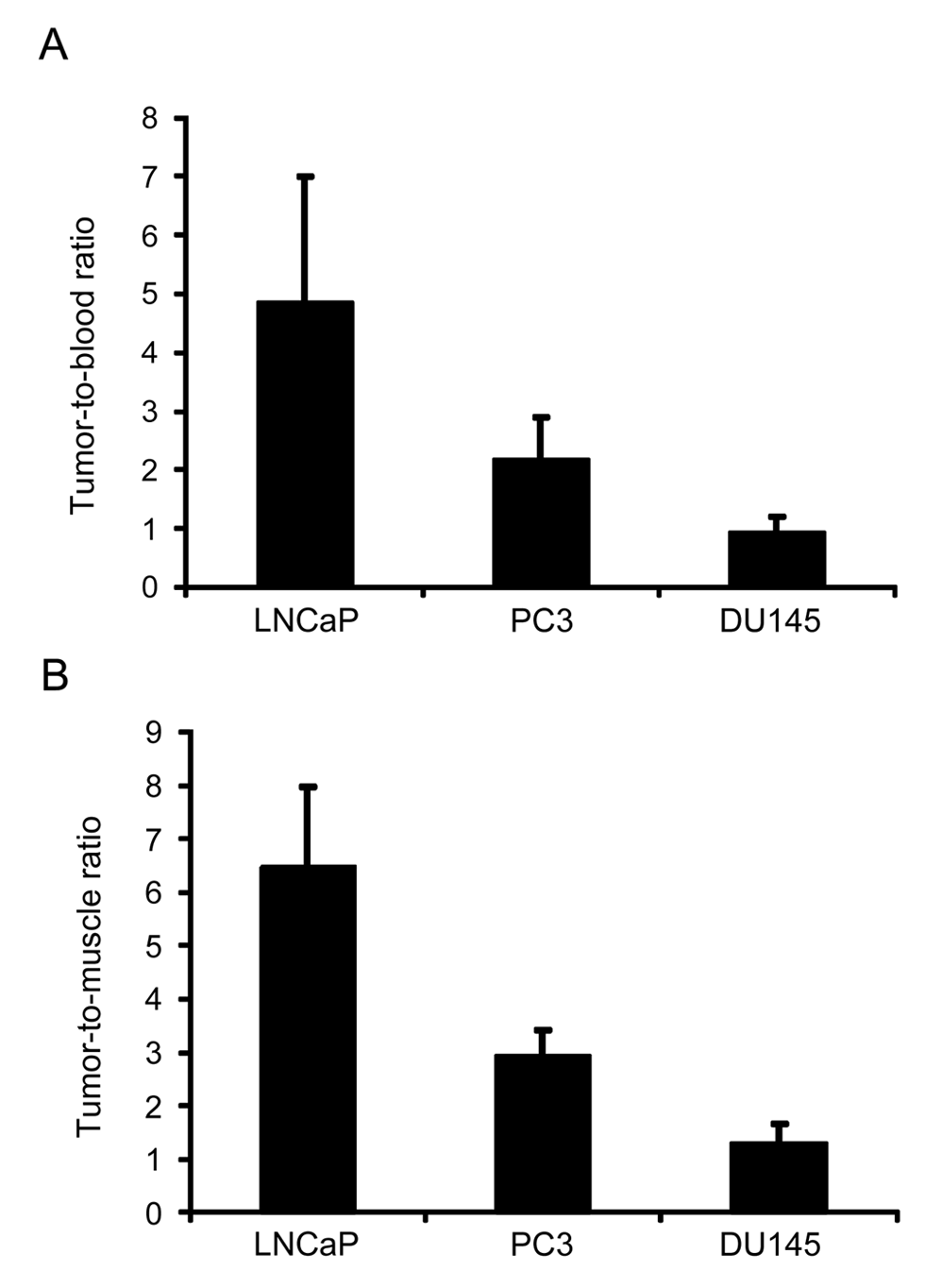

Supplement: Figure S2 — Tumor-to-blood and tumor-to-muscle ratios in biodistribution study. (TIF) [file pone.0064570.s002.tif]

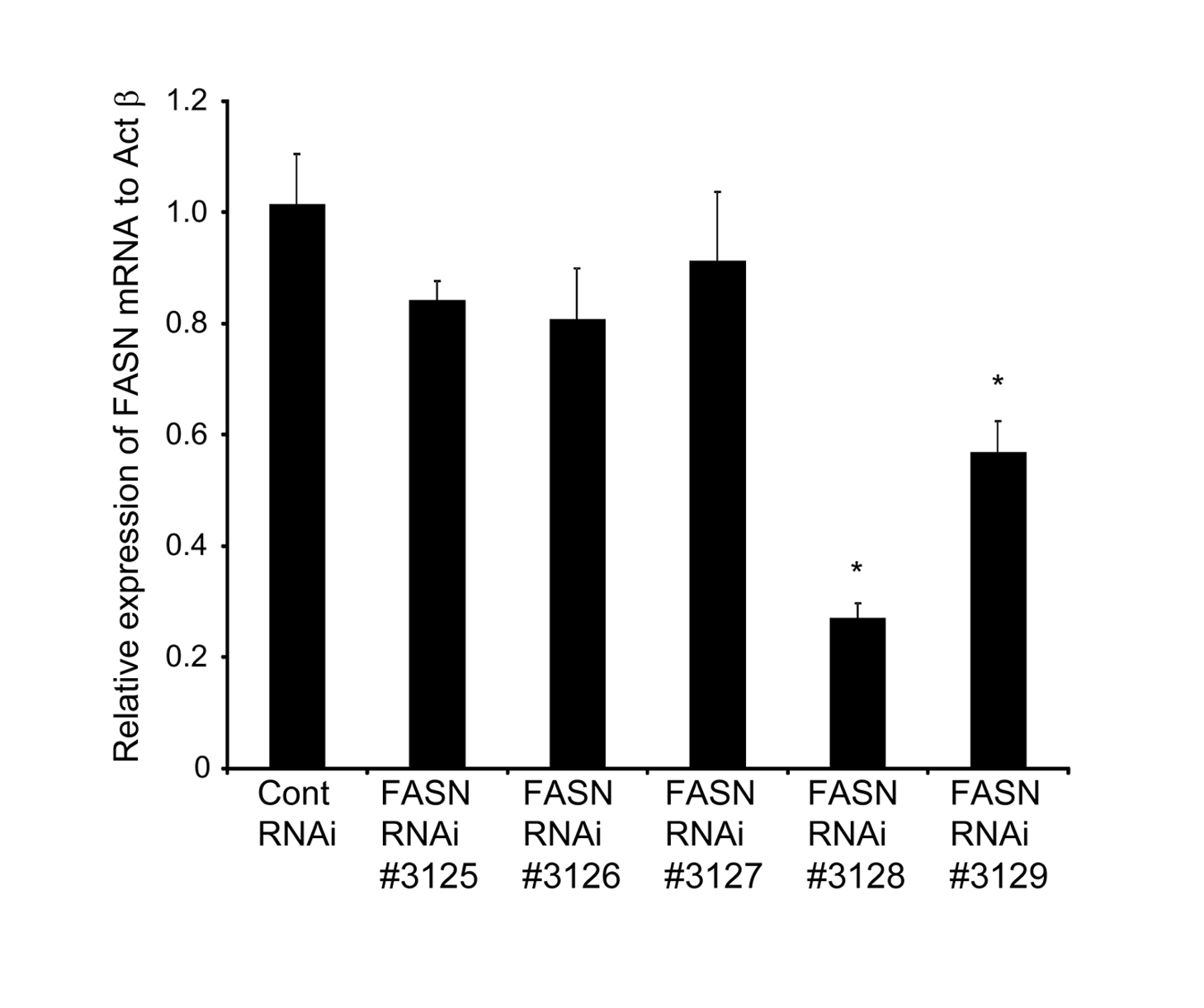

Supplement: Figure S3 — FASN mRNA expression in LNCaP cell lines transfected with shRNA against FASN. (TIF) [file pone.0064570.s003.tif]
