# Supplementary material for: Fatty Acid Synthase Is a Key Target in Multiple Essential Tumor Functions of Prostate Cancer: Uptake of Radiolabeled Acetate as a Predictor of the Targeted Therapy Outcome
Source: PLoS One. 2013 May 31;8(5):e64570. doi: 10.1371/journal.pone.0064570 (PMC3669310; doi:10.1371/journal.pone.0064570)
Supplement: Table S1 — Genes up-regulated by FASN inhibition with RNAi. (DOC) [file pone.0064570.s004.doc]

**Supplemental Table**

| **Table S1**  Genes up-regulated by FASN inhibition with RNAi* | | | |
| --- | --- | --- | --- |
| Gene Symbol | GeneName | Fold change | *P*-value |
| CA13 | carbonic anhydrase XIII | 11.199 | 2E-04 |
| TRPM8 | transient receptor potential cation channel, subfamily M, member 8 | 10.408 | 2E-04 |
| CAMK2N1 | calcium/calmodulin-dependent protein kinase II inhibitor 1 | 8.159 | 3E-05 |
| FAM5C | family with sequence similarity 5, member C | 6.797 | 8E-05 |
| S1PR3 | sphingosine-1-phosphate receptor 3 | 6.597 | 5E-05 |
| BANK1 | B-cell scaffold protein with ankyrin repeats 1 | 6.057 | 8E-05 |
| GSTA5 | glutathione S-transferase alpha 5 | 5.087 | 7E-05 |
| GUSBP1 | glucuronidase, beta pseudogene 1 | 3.292 | 2E-04 |
| GNAO1 | guanine nucleotide binding protein (G protein), alpha activating activity polypeptide O | 3.058 | 7E-05 |
| TRIM54 | tripartite motif-containing 54 | 2.955 | 2E-04 |
| CNBD1 | cyclic nucleotide binding domain containing 1 | 2.935 | 6E-05 |
| SALL4 | sal-like 4 (Drosophila) | 2.828 | 1E-04 |
| THBS1 | thrombospondin 1 | 2.812 | 2E-04 |
| AMACR | alpha-methylacyl-CoA racemase | 2.506 | 2E-04 |
| ENTPD2 | ectonucleoside triphosphate diphosphohydrolase 2 | 2.258 | 2E-04 |
| RHOB | ras homolog gene family, member B | 2.203 | 1E-04 |
| VSIG10L | V-set and immunoglobulin domain containing 10 like | 2.144 | 2E-04 |
| PHLDA2 | pleckstrin homology-like domain, family A, member 2 | 2.136 | 1E-04 |
| RTN4 | reticulon 4 | 2.066 | 7E-05 |
| C6orf147 | chromosome 6 open reading frame 147 | 2.065 | 5E-05 |
| LYPLAL1 | lysophospholipase-like 1 | 2.026 | 1E-04 |

*Genes up-regulated by FASN inhibition in FASN-RNAi 3128 cells in DNA microarray analysis. Genes significantly up-regulated by more than 2-fold (P < 0.05) are listed.
